# Medical Lectures in Buffalo

**Published:** 1865-10

**Authors:** 


### Medical Lectures in Buffalo.

The regular term of Lectures in the University of Buffalo commences November 4th. There are no changes in the Faculty so far as we are informed. Prof. White gives his course of Lectures upon Obsteterics and the Diseases of Women and Children, before leaving upon a tour of Europe. The preliminary term is largely attended, and there are flattering indications of a large class.

